# Efficient High-Resolution Sparse Channel Estimation Based on Temporal Correlation in MIMO-OFDM Systems

**DOI:** 10.3390/s26103136

**Published:** 2026-05-15

**Authors:** Hui Xie, Yide Wang, Guillaume Andrieux, Shaoyang Men

**Affiliations:** 1 School of Electronic Engineering, Tianjin University of Technology and Education, No.1310, Dagu South Road, Hexi District, Tianjin 300222, China; hui_xie_acad1981@163.com; 2 Nantes Université, CNRS, IETR, UMR 6164, F-44000 Nantes, France; yide.wang@univ-nantes.fr (Y.W.); guillaume.andrieux@univ-nantes.fr (G.A.); 3 School of Medical Information Engineering, Guangzhou University of Chinese Medicine, Guangzhou 510006, China

**Keywords:** multiple-input multiple-output orthogonal frequency division multiplexing (MIMO-OFDM), high-resolution sparse channel estimation, block-structured compressed channel sensing (CCS), temporal correlation, joint sparsity, prior delay support-aided delay tracking and block residual norm minimization (PDSA-DT-BRNM) algorithm

## Abstract

In this work, high-resolution sparse channel estimation in multiple-input multiple-output orthogonal frequency division multiplexing (MIMO-OFDM) systems is addressed. Firstly, a block-structured compressed channel sensing (CCS) model with high spectral efficiency and high delay resolution is constructed. Then, by fully exploiting the temporal correlation and joint sparsity of the channels, a novel two-stage prior delay support-aided delay tracking and block residual norm minimization (PDSA-DT-BRNM) algorithm is proposed. In the first stage, with a limited number of pilots for each antenna and the delay grids within the prior delay support, an efficient delay tracking and block norm minimization algorithm is put forward to choose the common delay grids and estimate each block gain iteratively. In the second stage, by comprehensively utilizing the intermediate channel estimation results of the first stage and the prior delay support, an optimized channel estimation strategy is developed based on the block residual norm minimization (BRNM) criterion. Simulation results and theoretical analysis show the effectiveness of the proposed channel estimation scheme in terms of channel estimation performance, spectral efficiency and computational complexity.

## 1. Introduction

Multiple-input multiple-output orthogonal frequency division multiplexing (MIMO-OFDM) technology has been extensively investigated and is widely regarded as a key technology underpinning future wireless communication systems, essentially due to its potential benefits, including high spatial diversity, multiplexing gains, and its ability to combat multipath fading [1,2]. However, obtaining accurate channel state information (CSI) remains a major challenge, primarily due to its substantial occupation of frequency resources and high computational cost.

The sparse common support (SCS) property [3], shared by most MIMO channels, is widely used for channel estimation in MIMO-OFDM systems. By employing the SCS characteristic in a MIMO system, numerous block sparse channel estimation methods based on compressed sensing (CS) theory [4] have been proposed [5,6,7,8,9,10]. Most of them assume that the channel is exactly sparse at discretized delays corresponding to integer multiples of the Nyquist period. However, such an assumption is not always true for practical channels, which may have taps with fractional delays known as non-sample spaced channels [11,12,13]. Discretized approximation of the practical channel at the Nyquist rate may lead to leakage effects [11]. In this case, virtual representation of channels with high resolution (sampling rate higher than the Nyquist rate) is widely considered in single-input and single-output (SISO) OFDM systems [12,13,14,15,16,17,18]. However, sparse channel reconstruction with high resolution requires a corresponding dictionary (i.e., the measurement matrix in CS), whose size increases dramatically with the oversampling factor *R*. This, in turn, leads to a substantial rise in computational complexity. To effectively reduce such computational complexity, refs. [13,14,15,16,17] propose efficient delay tracking and channel estimation methods in a SISO-OFDM system. Comparatively, in a MIMO-OFDM system, acquisition is more challenging due to the increased number of antennas (few available pilots) and the associated computational complexity.

Thanks to channel temporal correlation, multipath delays or multipath angles are likely to remain unchanged or partially unchanged within several adjacent OFDM symbols or consecutive frames [11,19,20,21,22,23]. Being aware of this, ref. [19] proposes a modified subspace pursuit (M-SP) algorithm for massive MIMO systems, which leverages prior support in the angle domain to achieve improved channel estimation performance while reducing pilot usage and computational complexity. By exploiting the temporal correlation, ref. [20] proposes a parametric channel estimation method in a MIMO-OFDM system based on the assumption that the channel sparse pattern is unchanged over several adjacent OFDM symbols. However, this assumption may not be suitable for time-varying channels, where the previously estimated CSI could be partially inaccurate or even outdated [9]. Nevertheless, it is still attractive to design effective channel estimation methods with high spectral efficiency and low computational complexity by exploring the channel temporal correlation.

In this work, high-resolution sparse channel estimation in MIMO-OFDM systems is investigated, where a block-structured compressed channel sensing (CCS) model with high spectral efficiency and high delay resolution is constructed, and a novel prior delay support-aided delay tracking and block residual norm minimization (PDSA-DT-BRNM) algorithm is proposed. Specifically, the contributions of this paper are summarized as follows.

- A block-structured CCS model with high resolution for non-sample spaced sparse channels is formulated. Different from the traditional high-resolution CCS model with the unique equispaced pilot arrangement strategy ($M\geq L_{cp}$) in SISO communication systems [14,15,16,17], our proposed CCS model for MIMO-OFDM systems employs a mixed pilot arrangement strategy. In this proposed model, the equispaced pilot arrangement ($M\geq L_{cp}$) is only used in the first OFDM symbol to obtain the prior delay estimate and a suboptimal pilot arrangement method with $M<L_{cp}$ [8] is adopted in the remaining subsequent OFDM symbols to effectively address the challenge of unaffordable pilot usage for channel estimation.
- In the first stage of the proposed PDSA-DT-BRNM algorithm, a novel prior delay support-aided delay tracking (PDSA-DT) algorithm is first employed to iteratively and effectively estimate the reference common delay grids (RCDGs). This is achieved by exploiting the delay grids obtained from the prior delay support and from the limited number of pilots. With the estimated RCDG, the intermediate delay support (Inter-DS) and the intermediate block gains (Inter-BGs) can be obtained using the BRNM algorithm.
- By fully exploiting the channel estimation results obtained from the first stage and the prior delay support, an optimized channel estimation strategy is developed in the second stage to strengthen the channel estimation performance based on the BRNM criterion.

The rest of the paper is organized as follows. The considered MIMO-OFDM system model is presented in Section 2. Section 3 describes the proposed method followed by the theoretical performance analysis in Section 4. Simulation results are provided in Section 5. Conclusions are drawn in Section 6.

## 2. System Model

### 2.1. MIMO-OFDM System Model

Consider a MIMO-OFDM system where the transmitter is equipped with $N_{t}$ transmit antennas and the receiver has a single antenna. Consider an OFDM system with *N* subcarriers, among which M(0<M≤N−1) subcarriers are pilots. The signal ${\tilde{x}}^{\left(i\right)}\left[n\right]$ is transmitted by the $i^{th}$$\left(0\leq i\leq N_{t}-1\right)$ antenna over the $i^{th}$ sparse channel with sparsity *K* (*K* ($K\ll L_{cp}$) non-zero channel taps [13,24]; $L_{cp}$ is the length of the cyclic prefix), which can be expressed as

(1) $$h^{\left(i\right)}\left[t\right]=\sum_{l=0}^{K-1}\alpha_{l}^{\left(i\right)}\delta \left(t-\tau_{l}^{\left(i\right)}T_{s}\right)$$

where $\alpha_{l}^{\left(i\right)}$, $\tau_{l}^{\left(i\right)}\left(0\leq \tau_{l}^{\left(i\right)}\leq L_{cp}-1\right)$ and $T_{s}$ are the complex gain, normalized delay of the $l^{th}$ tap of the $i^{th}$ channel and sampling interval respectively.

At the receiver, the received signal ${\tilde{y}}^{\left(i\right)}\left[n\right]$ can be written as

(2) $${\tilde{y}}^{\left(i\right)}\left[n\right]={\tilde{x}}^{\left(i\right)}\left[n\right]⊗h^{\left(i\right)}\left[n\right]+{\tilde{w}}^{\left(i\right)}\left[n\right]$$

where ${\tilde{w}}^{\left(i\right)}\left[n\right]$ is complex white Gaussian noise with zero mean and variance $\sigma_{t}^{2}$. The observed channel impulse response (CIR) of the $i^{th}$ antenna $h^{\left(i\right)}\left[n\right]$ can be characterized by a sinc-function given by [13]

(3) $$h^{\left(i\right)}\left[n\right]=\frac{1}{N}\sum_{l}\alpha_{l}^{\left(i\right)}e^{-j\frac{\pi }{N}\left(n+\left(N-1\right)\tau_{l}^{\left(i\right)}\right)}\frac{sin(\pi \tau_{l}^{\left(i\right)})}{sin\left(\frac{\pi }{N}\left(\tau_{l}^{\left(i\right)}-n\right)\right)}$$

For the non-sample spaced channel, $\tau_{l}^{\left(i\right)}$ is not an integer, which means that there exists power leakage of the $l^{th}$ tap. Increasing the delay resolution ($T_{s}^{\prime }=T_{s}/R$) is an effective way to reduce the leakage and achieve channel estimation with high precision.

Suppose that the normalized pilot matrix $diag\left[x^{\left(i\right)}\left(p^{\left(i\right)}\right)\right]\in C^{M\times M}$ is transmitted by the $i^{th}\left(i=0,1,\ldots ,N_{t}-1\right)$ antenna, where $p^{\left(i\right)}=\left\{p_{0}^{\left(i\right)},p_{1}^{\left(i\right)},\ldots ,p_{M-1}^{\left(i\right)}\right\},\left(0\leq p_{0}^{\left(i\right)}<p_{1}^{\left(i\right)},<\ldots ,<p_{M-1}^{\left(i\right)}\leq N-1\right)$ is the set of pilot patterns for the $i^{th}$ antenna. (Note: The pilot patterns used for different antennas are orthogonal to each other; i.e., $p^{\left(i\right)}\cap p^{\left(j\right)}=\emptyset ,i\neq j$). We have the corresponding received pilot vector due to the $i^{th}$ transmit antenna denoted as $y^{\left(i\right)}\left(p^{\left(i\right)}\right)\in C^{M\times 1}$. To simplify, we express the received pilot vector as $y^{\left(i\right)}=y^{\left(i\right)}\left(p^{\left(i\right)}\right)$ and the transmitted diagonal pilot matrix as $X=diag[x^{\left(i\right)}\left(p^{\left(i\right)}\right)]$. Then, the channel estimation model can be formulated by

(4) $$y^{\left(i\right)}=XF_{R}^{\left(i\right)}h_{R}^{\left(i\right)}+w^{\prime \left(i\right)},\;i=0,1,\ldots ,N_{t}-1$$

where $F_{R}^{\left(i\right)}\in C^{M\times (R(L_{cp}-1)+1)}$ is a dictionary matrix of the $i^{th}$ antenna with element $F_{R}^{\left(i\right)}\left(m,s\right)=e^{-j2\pi \frac{p_{m}^{\left(i\right)}{\tilde{\tau }}_{s}^{\left(i\right)}}{N}}$m∈{0,1,…,M−1}, $s\in \{0,1,\ldots ,R\left(L_{cp}-1\right)\}$, R=1 corresponds to the Nyquist baseband sampling factor while R≥2 is employed as the oversampling factor, ${\tilde{\tau }}_{s}^{\left(i\right)}\in T_{R}$, $T_{R}=\left\{0,\frac{1}{R},\frac{2}{R},\ldots ,L_{cp}-1\right\}$, $S=R(L_{cp}-1)+1$ is the size of the delay grids and $h_{R}^{\left(i\right)}\in C^{R(L_{cp}-1)+1}$ denotes the CIR vector of the $i^{th}$ antenna with oversampling factor *R*; $w^{\prime \left(i\right)}∼\mathrm{CN}\left(0,\sigma_{f}^{2}I_{M}\right)$ ($\sigma_{f}^{2}=N\sigma_{t}^{2}$) is the complex additive white Gaussian noise (AWGN) vector of the $i^{th}$ channel. By employing the least squares (LS) estimation method, we have the $i^{th}$ channel frequency response (CFR) $g^{\left(i\right)}\in C^{M\times 1}$ given by

(5) $$g^{\left(i\right)}=F_{R}^{\left(i\right)}h_{R}^{\left(i\right)}+w^{\left(i\right)},\;i=0,1,\ldots ,N_{t}-1$$

where $w^{\left(i\right)}$ is still a complex AWGN vector with same statistics as $w^{\prime \left(i\right)}$.

### 2.2. Pilot Arrangement for MIMO-OFDM System

**Figure 1:**
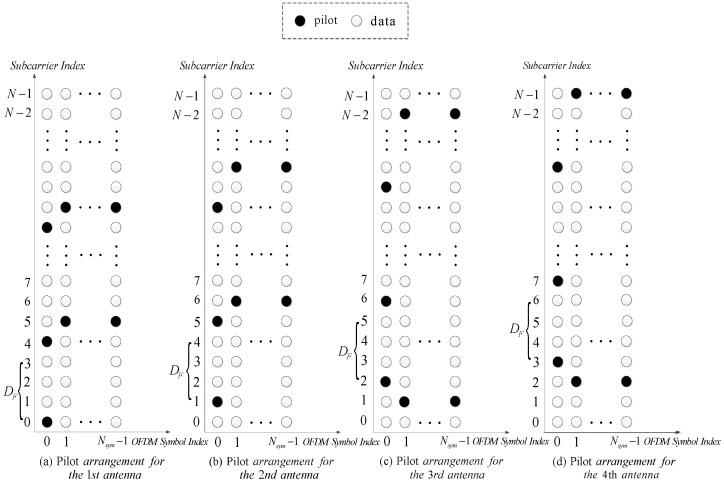
Pilot arrangements for MIMO-OFDM systems with $N_{t}=4$ antennas.

A mixed pilot arrangement strategy based on the combination of an equispaced pilot arrangement ($M\geq L_{cp}$) [14,15,16,24] and non-equispaced pilot arrangement $\left(M<L_{cp}\right)$ [7,8,13] is adopted for temporally correlated channel estimation in MIMO-OFDM systems as illustrated in Figure 1 with $N_{t}=4$ antennas. In the frequency domain, the pilots in the OFDM symbol with index 0 for the first antenna in Figure 1a, the second antenna in Figure 1b, the third antenna in Figure 1c and the fourth antenna in Figure 1d are equidistantly arranged with interval $D_{F}=4$. Additionally, to obtain the orthogonal pilot pattern for two arbitrary transmitted antennas mentioned above (i.e., $p^{\left(i\right)}\cap p^{\left(j\right)}=\emptyset ,\;0\leq i,j\leq N_{t}-1,\;i\neq j$), a unique initial index $I_{i}\left(0\leq i\leq N_{t}-1\right)$ for the $i^{th}$ antenna is introduced for $p_{0}^{\left(i\right)}\left(p_{0}^{\left(i\right)}=I_{i}\right)$. Finally, the $m^{th}$ pilot for the $i^{th}$ antenna is given by

(6) $$p_{m}^{\left(i\right)}=p_{0}^{\left(i\right)}+mD_{F}=I_{i}+mD_{F},\;m=0,1,\ldots ,M-1$$

The equispaced pilot arrangement for the $i^{th}$ antenna in (6) is essential for channel estimation with high precision. However, it is not spectrally efficient for a MIMO-OFDM system with $N_{t}$ antennas. Therefore, the equispaced pilot arrangement ($M\geq L_{cp}$) in the OFDM symbol with index 0 is utilized only to estimate the prior delay support, which is useful for the proposed PDSA-DT-BRNM algorithm to estimate channels with a limited number of pilots for each antenna ($M<L_{cp}$) in the following subsequent OFDM symbols indexed from 1 to $N_{sym}-1$.

Unlike the case where $M\geq L_{cp}$, the optimal pilot arrangement for $M<L_{cp}$ is generally non-equispaced and requires optimization. In SISO systems, the normalized mutual coherence of the measurement matrix is usually employed to optimize the pilot arrangement for the CCS. Traditionally, the normalized mutual coherence of $F_{R}^{\left(i\right)}$ is defined by

(7) $$\mu \left(F_{R}^{\left(i\right)}\right)=\max_{a,b\in T_{R},a\neq b}\frac{\left|\langle f_{a}^{\left(i\right)},f_{b}^{\left(i\right)}\rangle \right|}{∥f_{a}^{\left(i\right)}{∥}_{2}∥f_{b}^{\left(i\right)}{∥}_{2}}$$

where $f_{a}^{\left(i\right)}={\left[e^{\frac{-j2\pi p_{1}^{\left(i\right)}a}{N}},e^{\frac{-j2\pi p_{2}^{\left(i\right)}a}{N}},\ldots ,e^{\frac{-j2\pi p_{M}^{\left(i\right)}a}{N}}\right]}^{T},\;a\in T_{R}$ is a column of $F_{R}^{\left(i\right)}$. Considering that there are $N_{t}$ antennas and *M* orthogonal pilots are employed for each antenna, we have the following normalized mutual coherence minimization task derived from (7):

(8) $$\arg\min_{n(p^{\left(i\right)})=M}\max_{a,b\in T_{R},a<b}\left|\sum_{\gamma \in p^{\left(i\right)}}e^{-j2\pi \gamma (a-b)/N}\right|$$

where $n(p^{\left(i\right)})$ is the cardinality of $p^{\left(i\right)}$. To theoretically address (8), pilot arrangement subsets $p^{\left(i\right)},i=0,1,\ldots ,N_{t}-1$ should initially be obtained from the total subset Ω={0,1,…,N−1} with R≥2 and the constrained condition of $p^{\left(i\right)}\cap p^{\left(j\right)}=\emptyset$ (i≠j). Additionally, every pilot arrangement $p^{\left(i\right)},i=0,1,\ldots ,N_{t}-1$ subset should be optimized to make its corresponding $\left\{\mu \left(F_{R}^{\left(i\right)}\right)\right\},i=0,1,\ldots ,N_{t}-1$ as small as possible. However, in the case of R≥2, the computational complexity is *R* times higher than in the case where R=1. To efficiently solve (8) for practical applications, we consider the case of R=1 and adopt the effective pilot arrangement method proposed in [8]. The algorithm in [8] firstly generates a limited number of sets, which are random permutations of {1,2,…,N}, and extracts an order of ⌊N/M⌋ subsets with the same cardinality *M* from this limited number of sets. Each obtained ⌊N/M⌋ subset can be viewed as a group. For each group, the algorithm computes the normalized mutual coherence of each subset and obtains the $N_{t}$ number of subsets with the minimum normalized mutual coherence as well as their corresponding normalized mutual coherence vector. After finishing the above computation group by group, the algorithm tries to find the normalized mutual coherence vector whose largest element is minimum. Its corresponding subsets are then used as the suboptimal pilot arrangements for all multiple-antenna ports.

### 2.3. Block-Structured High-Resolution Compressed Channel Sensing in MIMO-OFDM Systems

For most wireless communication systems, the distance between any two antennas within either the transmit antenna array or the receive antenna array is significantly smaller than the transmission distance. The channels for different transmit antenna and receive antenna pairs share approximately the same scatters. Meanwhile, it is important to consider the oversampled resolvable distance $\frac{c}{RB}$ [3] (*c* is the speed of light and *B* is the bandwidth of the channels), which is the distance traveled by the electromagnetic wave in a time lapse within a normalized oversampling interval $\frac{1}{RB}$. If $d_{max}<\frac{c}{RB}$ ($d_{max}=N_{t}\frac{\lambda }{2}$; λ is the wavelength of the electromagnetic wave), the delays of different channels for any two pairs are approximately the same. Table 1 presents the parameters of bandwidth *B* and oversampled resolvable distance $\frac{c}{RB}$ for two different wireless communication systems. For example, in the 3GPP LTE wireless communication systems, $N_{t}=8$, $\frac{\lambda }{2}=0.075$ m (2 GHz, a popular carrier frequency in 3GPP LTE wireless communication systems), and $d_{max}=0.6$ m $<min\left\{\frac{c}{RB}\right\}=\frac{3\times 10^{8}\;m/s}{8\times 2\times 10^{7}\;\mathrm{Hz}}=1.875$ m. (Typically, we have R=2,4,8 in non-sample spaced channel estimation for a good tradeoff between the channel estimation performance and computational complexity [12,13,14,15,16,17]). A similar result can be observed in the IS-95 wireless communication systems. Therefore, it can be assumed that the delays of the $l^{th}$ path of the channels for different transmit antennas are the same, which are given by

(9) $$\tau_{l}^{\left(0\right)}=\tau_{l}^{\left(1\right)}=\ldots =\tau_{l}^{\left(N_{t}-1\right)}=\tau_{l}$$

where $\tau_{l}$ is the common delay of the $l^{th}$ tap. In other words, the channels of the MIMO-OFDM system tend to have a common delay set given by

(10) $$T^{\left(0\right)}=T^{\left(1\right)}=\ldots =T^{\left(N_{t}-1\right)}=T$$

where $T=\{\tau_{0},\tau_{1},\ldots ,\tau_{K-1}\}$. Hence, we have $h^{\left(i\right)}\left[\tau_{l}\right]\neq 0,\tau_{l}\in T,l\in \left\{0,1,\ldots ,K-1\right\}$. In order to explore the joint sparsity, a block-structured high-resolution compressed channel sensing model can be derived from (5) and given by

(11) $$z=\Phi_{R}c_{R}+\eta$$

where $z={\left[z_{0}^{T},z_{1}^{T},\ldots ,z_{M-1}^{T}\right]}^{T}\in C^{MN_{t}\times 1}$ is a block-structured CFR vector with its $m^{th}$ block element $z_{m}={\left[g^{\left(0\right)}\left(m\right),g^{\left(1\right)}\left(m\right),\ldots ,g^{\left(N_{t}-1\right)}\left(m\right)\right]}^{T}$(m=0,1,…,M−1); $c_{R}={\left[c_{R}^{T}\left(0\right),c_{R}^{T}\left(1\right),\ldots ,c_{R}^{T}\left(R\left(L_{cp}-1\right)\right)\right]}^{T}\in C^{\left(R\left(L_{cp}-1\right)+1\right)N_{t}\times 1}$ ($c_{R}\left(s\right)={\left[h_{R}^{\left(0\right)}\left(s\right),h_{R}^{\left(1\right)}\left(s\right),\ldots ,h_{R}^{\left(N_{t}-1\right)}\left(s\right)\right]}^{T}$ ($s=0,1,\ldots ,R(L_{cp}-1)$)) is approximately a block-structured sparse channel vector; and $\eta ={\left[\eta_{0}^{T},\eta_{1}^{T},\ldots ,\eta_{M-1}^{T}\right]}^{T}\in C^{MN_{t}\times 1}$ is a block-structured Gaussian noise vector, the $m^{th}$ block element of which is $\eta_{m}={\left[w^{\left(0\right)}\left(m\right),w^{\left(1\right)}\left(m\right),\ldots ,w^{\left(N_{t}-1\right)}\left(m\right)\right]}^{T}$(m=0,1,…,M−1). $\Phi_{R}\in C^{MN_{t}\times \left(R\left(L_{cp}-1\right)+1\right)N_{t}}$ is the block-structured high-resolution dictionary matrix generated from a $\left(M\times \left(R\left(L_{cp}-1\right)+1\right)\right)$ matrix with its $m^{th}$ row and $s^{th}$ column entry given by $diag[F_{R}^{\left(0\right)}\left(m,s\right),F_{R}^{\left(1\right)}\left(m,s\right),\ldots ,F_{R}^{\left(N_{t}-1\right)}\left(m,s\right)]$ (m=0,1,…,M−1, $s=0,1,\ldots ,R(L_{cp}-1)$). $\Phi_{R}$ can also be presented by the following block form:

(12) $$\begin{array}{c} \Phi_{R}\;=\;[\underset{\Phi_{0}}{\underset{︸}{\phi_{0},\phi_{1},\ldots ,\phi_{N_{t}-1}}},\underset{\Phi_{\frac{1}{R}}}{\underset{︸}{\phi_{N_{t}},\phi_{N_{t}+1},\ldots ,\phi_{2N_{t}-1}}},\\ \ldots ,\underset{\Phi_{L_{cp}-1}}{\underset{︸}{\phi_{R(L_{cp}-1)N_{t}},\phi_{R(L_{cp}-1)N_{t}+1},\ldots ,\phi_{R(L_{cp}-1)N_{t}+N_{t}-1}}}] \end{array}$$

where $\phi_{j}\in C^{MN_{t}\times 1}\left(0\leq j\leq R\left(L_{cp}-1\right)N_{t}+N_{t}-1\right)$ is the $j^{th}$ column of $\Phi_{R}$; $\Phi_{\tau }\left(\tau \in T_{R}\right)$ is the block sub-bases of $\Phi_{R}$ with delay grid τ.

## 3. Proposed PDSA-DT-BRNM Algorithm

In a MIMO-OFDM system, the path delays vary much more slowly than the path gains due to temporal correlation; therefore, the delays of the channel paths are likely to remain unchanged or partially unchanged over several adjacent OFDM symbols or consecutive frames [11,19,20,21,22]. In this case, it is reasonable to develop algorithms to estimate the delays by utilizing prior delay support within the interval of several adjacent OFDM symbols or consecutive frames.

### 3.1. First Stage of the Proposed PDSA-DT-BRNM Algorithm

#### 3.1.1. Prior Delay Support-Aided Delay Tracking (PDSA-DT) Algorithm

Considering the block-structured high-resolution compressed channel sensing model in (11), the solution to obtain an estimate of the block channel ${\hat{c}}_{R}$ can be formulated as the following constrained $l_{2}$ minimization problem:

(13) $${\hat{c}}_{R}=\arg\min_{c}{∥z-\Phi_{R}c∥}_{2}\;s.t\;∥supp{(c)∥}_{0}=K$$

where c is a block-structured vector with block sparsity *K* and $supp(c)=\left\{s:{∥c\left[s\right]∥}_{2}\neq 0\right\}$ is the support of c. However, the optimal solution to (13) is practically infeasible due to its prohibitively high computational cost. The block orthogonal matching pursuit (BOMP) algorithm provides a suboptimal but computationally feasible solution to (13) [25] and is widely used in the field of block-structured sparse channel estimation [5,6]. To clearly illustrate the proposed method and the compared BOMP method, block-structured multipath channels with a block sparsity of K=1 are considered (no intercarrier interference (ICI) and noise). The common delay estimated by BOMP with oversampling factor *R*, ${\hat{\tau }}_{0,BOMP}$, can be obtained by

(14) $${\hat{\tau }}_{0,BOMP}=\arg\max_{\tau \in T_{R}}{∥\Phi_{\tau }^{H}z∥}_{2}$$

**Figure 2:**
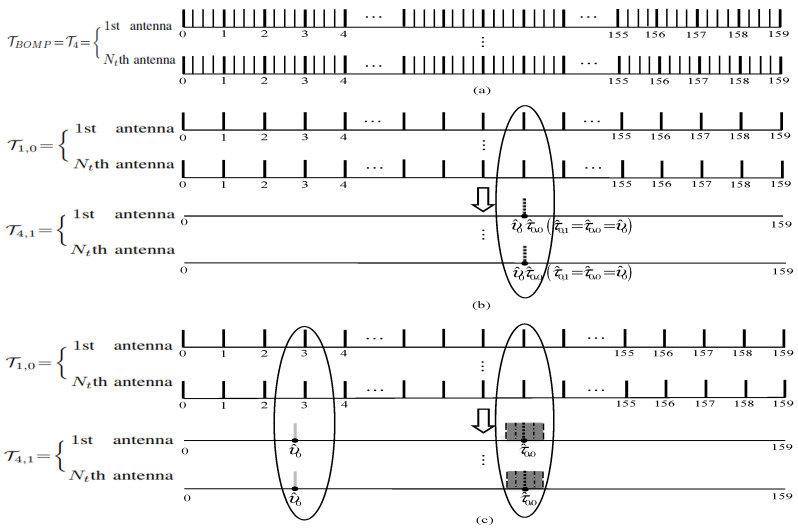
Common delay grids of the BOMP algorithm (**a**); common delay grids of the proposed PDSA-DT algorithm with the first case (**b**); common delay grids of the proposed PDSA-DT algorithm with the second case (**c**).

The computational complexity of the block delay tracking in (14) is important, especially when large *R* is required to obtain high channel estimation precision and big $N_{t}$ is required for efficient wireless communication transmission. An example of the common delay grids of the BOMP algorithm with R=4, K=1 and $L_{cp}=160$ is illustrated in Figure 2a; the size of its common delay grids is ${∥T_{R}∥}_{0}=R\left(L_{cp}-1\right)+1=637$. If a more general wireless communication scenario with a block sparsity of K>1 is considered, the total computational complexity will be significant, which may considerably reduce its attractiveness for practical wireless communication systems.

In order to develop a more efficient channel estimation method, we firstly obtain a rough common delay estimate ${\hat{\tau }}_{0,0}$ using the roughest common delay grid with an oversampling factor of R=1:

(15) $${\hat{\tau }}_{0,0}=\arg\max_{\tau \in T_{1,0}}{∥\Phi_{\tau }^{H}z∥}_{2}$$

where ${\hat{\tau }}_{0,0}$ is the rough estimate of $\tau_{0}$ and $T_{1,0}=T_{1}$. According to the properties of ${∥\Phi_{\tau }^{H}z∥}_{2}=\sqrt{\sum_{i=0}^{N_{t}-1}{|h_{0}^{\left(i\right)}|}^{2}}f\left(\Delta \tau \right)$ ($f\left(\Delta \tau \right)=\frac{\left|sin(\pi \Delta \tau )\right|}{\left|sin(\pi \Delta \tau /M)\right|}$, $\Delta \tau =\tau_{0}-\tau$, $\Delta \tau \in [-L_{cp}+1,L_{cp}-1]$, $\tau \in T_{1,0}$) and those of f(Δτ) in Appendix A, ${∥\Phi_{\tau }^{H}z∥}_{2}$ shares two major properties with f(Δτ). (1) f(Δτ) monotonously increases and decreases within the ranges $\left[-\frac{1}{2},0\right)$ and $\left(0,\frac{1}{2}\right]$ respectively. (2) For the ranges $\Delta \tau \in \left[-L_{cp}+1,-\frac{1}{2}\right)$ and $\Delta \tau \in \left(\frac{1}{2},L_{cp}-1\right]$, we have $f\left(\Delta \tau \right)<f\left(-\frac{1}{2}\right)$ and $f\left(\Delta \tau \right)<f\left(\frac{1}{2}\right)$ respectively. Therefore, it is reasonable to consider the boundary of error of the rough common delay estimate $\Delta \tau =\left|{\hat{\tau }}_{0,0}-\tau_{0}\right|\leq \frac{1}{2}$. With the rough delay estimate ${\hat{\tau }}_{0,0}$, the properties of function f(Δτ) have been well exploited to obtain finer resolution delay estimation in the SISO-OFDM system ($N_{t}=1$) [14,15,16,17]. However, the algorithms in [14,15,16,17] are under the condition of an equispaced pilot arrangement and $M>L_{cp}$, which are not suitable for the MIMO-OFDM system, since the total number of pilots is proportional to $N_{t}$.

Thanks to the temporal correlations, the delay grids within the prior delay support can be explored to improve the channel estimation efficiency and performance. To facilitate the access and usage of the delay grids within the prior delay support, the prior delay estimation support vector can be set as $\hat{\Upsilon }$ and $\hat{\Upsilon }=\left\{{\hat{\upsilon }}_{0}\right\}\subset T_{R}$ (K=1), where ${\hat{\upsilon }}_{0}$ is the element of $\hat{\Upsilon }$. $\hat{\Upsilon }$ can be estimated by the BOMP algorithm with $M(M\geq L_{cp}$) equispaced pilots in the OFDM symbol with index 0, which is presented in Section 2.2. Here, it is worth stating that although the pilot ratio (PR=M/N) and the computational complexity in the OFDM symbol with index 0 are comparatively higher, the average pilot ratio $PR_{ave}$ and average computational complexity remain comparatively low when considering a large number of subsequent OFDM symbols (big $N_{sym}$) discussed respectively in Section 4.1 and Section 4.2. More important, by doing so, the estimated prior delay support vector $\hat{\Upsilon }$ can be shared by the large number of subsequent OFDM symbols or consecutive frames to estimate channels with high precision for MIMO transmission owing to the common delay support shared by different antennas. Here, the time-varying wireless communication scenario is considered where the delays of channels within several adjacent OFDM symbols or consecutive frame intervals may differ with some probability [9]. Therefore, taking the only two available estimated delays, the prior-estimated delay ${\hat{\upsilon }}_{0}$ and roughly estimated delay ${\hat{\tau }}_{0,0}$, into account, we have the following two cases:

1. In the majority of cases, the tolerable prior delay estimation error is expressed by $\left|{\hat{\upsilon }}_{0}-{\hat{\tau }}_{0,0}\right|\leq \frac{1}{2R},{\hat{\upsilon }}_{0}\in \hat{\Upsilon }$ in Figure 2b;

2. In the minority of cases, the intolerable prior delay estimation error is expressed by $\left|{\hat{\upsilon }}_{0}-{\hat{\tau }}_{0,0}\right|>\frac{1}{2R}$ in Figure 2c.

For convenient illustration and comparison, both Figure 2b,c share the same *R*, *K* and $L_{cp}$ with Figure 2a.

(1) $\left|{\hat{\upsilon }}_{0}-{\hat{\tau }}_{0,0}\right|\leq \frac{1}{2R}$

This situation means that the channel does not change significantly; we firstly have ${\hat{\tau }}_{0,0}={\hat{\upsilon }}_{0}$ due to the constrained delay resolution 1/R for channel estimation in the MIMO-OFDM system. Then, a local estimated delay ${\hat{\tau }}_{0,1}$ can be obtained by considering ${\hat{\tau }}_{0,1}={\hat{\tau }}_{0,0}={\hat{\upsilon }}_{0}$. Finally, we get the local finer resolution common delay set $T_{R,1}$ in Figure 2b expressed by

(16) $$T_{R,1}=\left\{{\hat{\tau }}_{0,1}\right\}$$

Since the real delay $\tau_{0}$ is unknown, the delay ${\hat{\tau }}_{0,1}$ in $T_{R,1}$ can be used for the reference common delay grid (RCDG), which is given by

(17) $$T_{RCDG}=T_{R,1}=\left\{{\hat{\tau }}_{0,1}\right\}$$

(2) $\left|{\hat{\upsilon }}_{0}-{\hat{\tau }}_{0,0}\right|>\frac{1}{2R}$

According to the properties of the function f(Δτ) in Appendix A, no matter what the values are for $\frac{1}{2R}$, ${\hat{\upsilon }}_{0}$ cannot support the channel estimation with high precision (the channel has been changed significantly). In this case, local finer common delay expansion $T_{R,1}$ with R≥2 should be adopted for tracking the finer resolution delay, which can be expressed by [13]

(18) $$\begin{array}{cc} T_{R,1}= & \{{\hat{\tau }}_{0,0}-\frac{1}{2},{\hat{\tau }}_{0,0}-\frac{1}{2}+\frac{1}{R},\ldots ,{\hat{\tau }}_{0,0},\ldots ,\\ & {\hat{\tau }}_{0,0}+\frac{1}{2}-\frac{1}{R},{\hat{\tau }}_{0,0}+\frac{1}{2}\} \end{array}$$

With the delay grids within the shadow area $T_{R,1}$ in Figure 2c, the RCDG of the second case can be obtained by

(19) $$T_{RCDG}=sup\left({\left({∥\Phi_{R,T_{R,1}}^{H}z∥}_{2}\right)}_{D}\right)$$

where $T_{RCDG}$ includes the block position indices of the *D* largest block coherence indices of $\Phi_{R,T_{R,1}}^{H}z$ (typically, D=2,3).

#### 3.1.2. Block Residual Norm Minimization (BRNM) Algorithm

With the RCDG in the two cases in (17) and (19), the block-structured sparse channel estimation can be obtained by realizing the following block residual norm minimization (BRNM) algorithm:

(20) $$\left[{\hat{c}}_{0},\;{\hat{\tau }}_{0}\right]=\arg\min_{c^{\prime },\tau^{\prime }\in T_{RCDG}}{∥z-{\hat{\Phi }}_{\tau^{\prime }}c^{\prime }∥}_{2}$$

In the first case, ${∥T_{RCDG}∥}_{0}=1$, one time block residual norm computation is needed, while in the second case, two or three block residual norm computations are required for high channel estimation precision. By exploiting the prior delay grids within the prior delay support, the largest proportion is occupied by the first case where the prior-estimated common delay grid ${\hat{\upsilon }}_{0}$ with high precision is used; therefore, the average number of block residual norm computations is less than three when estimating one block channel tap, which is helpful for the reduction in total computational complexity.

### 3.2. Second Stage of the Proposed PDSA-DT-BRNM Algorithm for Block-Structured High-Resolution Sparse Channels

For the general wireless communication scenario where K>1, the proposed PDSA-DT algorithm in Section 3.1.1 and the proposed BRNM algorithm in Section 3.1.2 are extended to iteratively estimate the intermediate delay support (Inter-DS) $T_{Inter-DS}$ and the intermediate block gains (Inter-BGs) $c_{Inter-BG}$, which are given in Algorithm 1. After *K* ($K={∥\hat{\Upsilon }∥}_{0}$, $\hat{\Upsilon }=\left\{{\hat{\upsilon }}_{0},\ldots ,{\hat{\upsilon }}_{l},\ldots {\hat{\upsilon }}_{K-1}\right\}\subset T_{R}$) iterations, the obtained intermediate delay support (Inter-DS) $T_{Inter-DS}$ and the intermediate block gains (Inter-BGs) $c_{Inter-BG}$ are still not optimal due to the insufficient number of pilots for each antenna.

In order to obtain optimal channel estimation results, a new strategy is put forward to optimize the channel estimation by exploiting the intermediate delay support (Inter-DS) $T_{Inter-DS}$ of the first stage and the prior delay support $\hat{\Upsilon }$, which is given by

(21) $$\begin{array}{c} \left[{\hat{c}}_{PDSA-DT-BRNM},\;T_{PDSA-DT-BRNM}\right]\\ =\arg\min_{c,T\in T_{RCDS}}{∥z-{\hat{\Phi }}_{R,T}c∥}_{2} \end{array}$$

where $T_{RCDS}=\left[T_{Inter-DS}\;\hat{\Upsilon }\right]$ is the reference common delay support. In (21), the delay support of the proposed PDSA-DT-BRNM method $T_{PDSA-DT-BRNM}$ and the block gains of the proposed PDSA-DT-BRNM method ${\hat{c}}_{PDSA-DT-BRNM}$ are optimized by considering either $T_{Init-DS}$ or $\hat{\Upsilon }$ from $T_{RCDS}$ based on the block residual norm minimization (BRNM) criterion.
**Algorithm 1** Proposed PDSA-DT-BRNM algorithm
Input:
(1) Initial residual vector $r_{-1}=z$; (2) Oversampling factor *R*; (3) Prior delay support vector obtained by BOMP $\hat{\Upsilon }=\left\{{\hat{\upsilon }}_{0},{\hat{\upsilon }}_{1},\ldots {\hat{\upsilon }}_{K-1}\right\}$; (4) Estimated channel block sparsity $K={∥\hat{\Upsilon }∥}_{0}$; (5) Size of the set of reference common delay grids in the second case *D*; (6) Initial set of intermediate delay support $T_{Inter-DS}\left(-1\right)=\emptyset$; (7) Initial matrix with the selected block bases ${\hat{\Phi }}_{-1}^{\prime }=\emptyset$; (8) Initial the tap number l=0. Main body: 1: while (l<K) 2: ${\hat{\tau }}_{l,0}=\arg\min_{\tau \in T_{1,0}}{∥\Phi_{1,\tau }^{H}z∥}_{2}$; % Get the initial maximum block coherence; 3: if $min\left|{\hat{\upsilon }}_{s}-{\hat{\tau }}_{l,0}\right|\leq \frac{1}{2R},\;{\hat{\upsilon }}_{s}\in \hat{\Upsilon },\;s\in \left\{0,1,2,\ldots ,K-1\right\}$ 4: $T_{RCDG}\left(l\right)=\left\{{\hat{\tau }}_{l,1}\right\}$$\left({\hat{\tau }}_{l,1}={\hat{\tau }}_{l,0}={\hat{\upsilon }}_{l},\;{\hat{\upsilon }}_{l}=\arg\min_{{\hat{\upsilon }}_{s}\in \hat{\Upsilon }}\left|{\hat{\upsilon }}_{s}-{\hat{\tau }}_{l,0}\right|\right)$ 5: elseif $min\left|{\hat{\upsilon }}_{s}-{\hat{\tau }}_{l,0}\right|>\frac{1}{2R}$ 6: $T_{R,1}\left(l\right)=\left\{{\hat{\tau }}_{l,0}-\frac{1}{2},{\hat{\tau }}_{l,0}-\frac{1}{2}+\frac{1}{R},\ldots ,{\hat{\tau }}_{l,0},\ldots ,{\hat{\tau }}_{l,0}+\frac{1}{2}-\frac{1}{R},{\hat{\tau }}_{l,0}+\frac{1}{2}\right\}$ 9: $T_{RCDG}\left(l\right)=sup\left({\left({∥\Phi_{R,T_{R,1}\left(l\right)}^{H}z∥}_{2}\right)}_{D}\right)$ 10: end 11: $\left[{\hat{c}}_{l},\;{\hat{\tau }}_{l}\right]=\arg\min_{c^{\prime },\tau^{\prime }\in T_{RCDG}\left(l\right)}{∥z-\left[{\hat{\Phi }}_{l-1}^{\prime }\;{\hat{\Phi }}_{\tau^{\prime }}\right]c^{\prime }∥}_{2}$ 12: $T_{Inter-DS}\left(l\right)=\left[T_{Inter-DS}\left(l-1\right)\;{\hat{\tau }}_{l}\right]$ 13: ${\hat{\Phi }}_{l}^{\prime }=\left[{\hat{\Phi }}_{l-1}^{\prime }\;\Phi_{{\hat{\tau }}_{l}}\right]$ 14: $r_{l}=z-{\hat{\Phi }}_{l}^{\prime }{\hat{c}}_{l}$ 15: if l==K−1 16: $\left[{\hat{c}}_{PDSA-DT-BRNM},\;T_{PDSA-DT-BRNM}\right]$ $=\arg\min_{c,T\in T_{RCDS}}{∥z-{\hat{\Phi }}_{R,T}c∥}_{2}$
$\left(T_{RCDS}=\left[T_{Inter-DS}\;\hat{\Upsilon }\right]\right)$ 17: l=l+1 18: end Output: ${\hat{c}}_{PDSA-DT-BRNM}=\hat{c}\left(T_{PDSA-DT-BRNM}\right)$ % Estimated the block structured high resolution CIR with PDSA-DT-BRNM method


## 4. Performance Analysis of the Proposed PDSA-DT-BRNM Algorithm

Here, we carry out the performance analysis of the proposed PDSA-DT-BRNM algorithm from the perspective of spectral efficiency and computational complexity.

### 4.1. Spectral Efficiency

It is known that the equispaced pilot arrangement is optimal for the case of $M\geq L_{cp}$ in a SISO system [13,14,15,16,17]. Considering our MIMO wireless communication scenario, its communication channels can be divided into $N_{t}$ independent SISO channels. Therefore, at least $N_{t}L_{cp}$ pilots are required to realize an optimal channel estimation in one OFDM symbol. However, this is not spectrally efficient for a MIMO-OFDM system. Thanks to the pilot arrangement algorithm in Section 2.2, the proposed algorithm only requires $N_{t}M_{0}$ pilots for the OFDM symbol with index 0 and $N_{t}M$ pilots for OFDM symbols with indices $1,2,\ldots ,N_{sym}-1$. For example, consider a MIMO-OFDM system with parameters N=4096, $N_{t}=8$, $M_{0}=256$, M=36, $L_{cp}=256$ and $N_{sym}=32$. The conventional equispaced pilot arrangement requires at least $N_{t}L_{cp}=2048$ pilots, which occupies 50% of the total subcarriers for each OFDM symbol; therefore, it has the average pilot ratio $PR_{ave}=50\%$, while for the proposed PDSA-DT-BRNM method, $PR_{ave}=\frac{N_{t}M_{0}+N_{t}M\left(N_{sym}-1\right)}{NN_{sym}}=8.37\%$. If $N_{sym}$ is sufficiently large, we have $PR_{ave}\approx N_{t}M/N\approx 7.03\%$, which means the increased pilot number in the OFDM symbol with index 0 can be omitted.

### 4.2. Computational Complexity

Table 2 compares the computational complexity of the proposed PDSA-DT-BRNM algorithm and the BOMP algorithm, where the BOMP algorithm is adopted to obtain the prior delay support from the OFDM symbol with index 0. Based on the previous analysis, this algorithm uses $R(L_{CP}-1)+1$ common delay grids. Consequently, its computational complexity is $O(M_{0}N_{t}\left(R\left(L_{CP}-1\right)+1\right)K)$.

The computational complexity of the proposed PDSA-DT-BRNM method primarily stems from its two-stage processing architecture, which is applied to each OFDM symbol with indices $0,1,\ldots ,N_{sym}-1$. The first stage of this process combines the PDSA-DT and BRNM algorithms. In the proposed PDSA-DT algorithm, there are two cases for each iteration. In the first case, the proposed PDSA-DT algorithm considers $L_{cp}$ common delay grids; correspondingly, it requires $L_{cp}$ block coherence computations, while in the second case, it has $L_{cp}+R$ block coherence computations. Therefore, considering block-structured sparse channels with the block sparsity *K*, the total complexity of the proposed PDSA-DT algorithm for the first case and the second case is $O(MN_{t}L_{cp}K)$ and $O(MN_{t}\left(L_{cp}+R\right)K)$ respectively. In the proposed BRNM algorithm, only one pseuoinverse and residual update computation is required for the first case; comparatively, in the second case, two or three pseuoinverse and residual updates computations are required. Therefore, the computational complexity for either case is less than $O(MN_{t}\left(L_{cp}+R\right)K)$. In the second stage, the optimized channel estimation strategy has a computational complexity less than $O(MN_{t}\left(L_{cp}+R\right)K)$. Based on the above-detailed analysis, the total computational complexity of the proposed PDSA-DT-BRNM method is $O(3MN_{t}\left(L_{cp}+R\right)K)$.

With the computational complexity analysis of different OFDM symbols mentioned above, the average computational complexity for the proposed PDSA-DT-BRNM algorithm is $O(\frac{M_{0}N_{t}\left(R\left(L_{CP}-1\right)+1\right)K+3MN_{t}\left(L_{cp}+R\right)K\left(N_{sym}-1\right)}{N_{sym}})$. Similarly to the analysis of the spectral efficiency, the average computational complexity of the proposed PDSA-DT-BRNM method approximates to $O(3MN_{t}\left(L_{cp}+R\right)K)$ when $N_{sym}$ is sufficiently large.

The BOMP algorithm has a computational complexity of $O(MN_{t}\left(R\left(L_{CP}-1\right)+1\right)K)$ for each OFDM symbol.

## 5. Simulation Results and Discussion

To evaluate the channel estimation performance of the proposed PDSA-DT-BRNM algorithm, simulations are conducted in MATLAB R2015a in this section. We consider a MIMO-OFDM system with $N_{t}=1,2,4$ or 8 antennas for the transmitter. The system has N=4096 subcarriers, among which 256 pilot subcarriers and 36 pilot subcarriers are allocated to each transmitted antenna for the OFDM symbol with index 0 and the remaining subsequent OFDM symbols with indices $1,2,\ldots ,N_{sym}-1=31$ ($N_{sym}=32$) respectively. (Note: The acquisition of the 36-pilot-subcarrier arrangement for $N_{t}=1,2$ or 4 antennas is realized by randomly extracting one, two or four pilot arrangement vectors from the pilot arrangement matrix with $N_{t}=8$ obtained from the pilot arrangement algorithm in Section 2.2). Quadrature Phase Shift Keying (QPSK) is employed for modulation. The sampling frequency is $f_{s}=1/T_{s}=30.72$ MHz and the cyclic prefix is $L_{cp}=160$ [16]. In our simulations, the COST 207 Typical Urban six-path (TU6) channel is adopted whose power delay profile is given in Table 3 [16,26,27]. The delay of each path of the COST 207 TU6 channel is shared by most antennas and the multipath amplitude of all antennas is subjected to a Rayleigh distribution. Additionally, the oversampling factor is set to be R=2,4 or 8. Moreover, we consider the time-varying communication scenario where the prior delay support Υ in the first OFDM symbol may differ from the actual delay support T in the remaining subsequent OFDM symbols with 25% probability. Specifically, the prior delay support Υ may have one or two arbitrary delays $\upsilon_{j}$ uniformly distributed in $\left(0,L_{cp}-1\right)$ with the additional constraint $|\upsilon_{j}-\tau_{i}|\geq 0.5$ (the deviation bound of $\upsilon_{j}$ and an arbitrary delay element $\tau_{i}$(0≤i≤K−1)) in T are no less than 0.5) [18]. $\hat{\Upsilon }$ is estimated by the BOMP method with the channel sparsity *K*.

The channel estimation performance is evaluated by the normalized mean square error (NMSE) given by

(22) $$NMSE=\frac{1}{N_{sym}N_{t}}\sum_{j=0}^{N_{sym}-1}\sum_{i=0}^{N_{t}-1}\frac{∥{\hat{g}}_{j}^{\left(i\right)}-g_{j}^{\left(i\right)}{∥}_{2}^{2}}{∥g_{j}^{\left(i\right)}{∥}_{2}^{2}}$$

where $g_{j}^{\left(i\right)}$ is the CFR of the $i^{th}$ ($0\leq i\leq N_{t}-1$, $0\leq j\leq N_{sym}-1$) antenna and $j^{th}$ OFDM symbol; ${\hat{g}}_{j}^{\left(i\right)}$ is the estimate of $g_{j}^{\left(i\right)}$.

**Figure 3:**
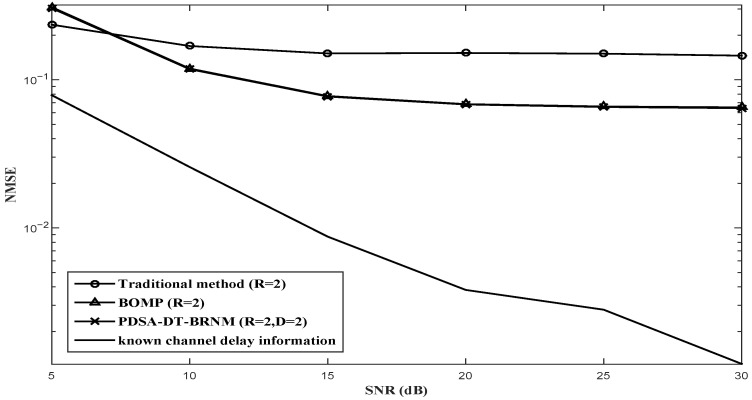
NMSE performance comparison of different algorithms with $N_{t}=2$ and R=2.

**Figure 4:**
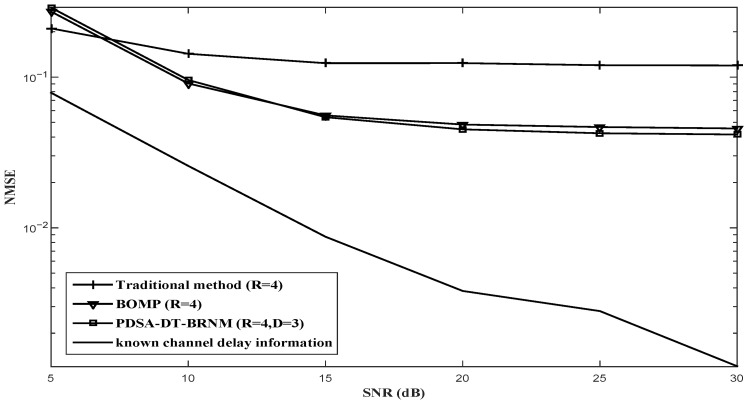
NMSE performance comparison of different algorithms with $N_{t}=2$ and R=4.

**Figure 5:**
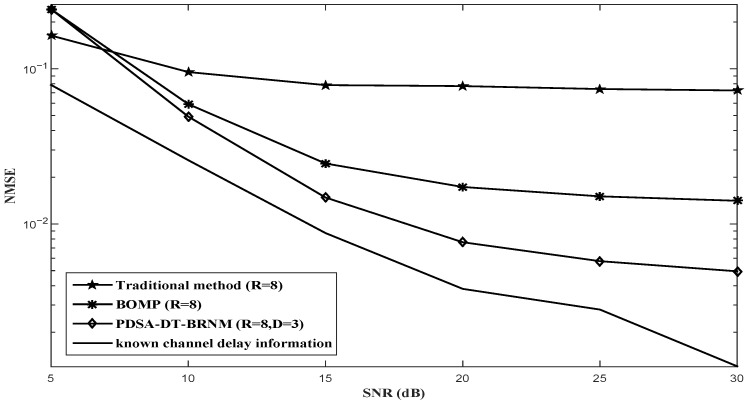
NMSE performance comparison of different algorithms with $N_{t}=2$ and R=8.

The NMSE performance comparisons for the proposed PDSA-DT-BRNM (D = 2, D = 3) algorithm, BOMP algorithm and other channel estimation algorithms with $N_{t}=2$ and different *R* (R=2, R=4 or R=8) are illustrated in Figure 3, Figure 4 and Figure 5 respectively. It can be observed that the proposed PDSA-DT-BRNM method outperforms the classical BOMP algorithm with the same oversampling factor R=2,4 or 8 and their performance gap monotonously increases with *R*. Additionally, the performance of the traditional method with R=2,4 or 8 (which uses the prior delay support directly) is poor mainly due to the effect of the partial elements with intolerable error. The known channel delay information method achieves the best performance, which is used for reference.

**Figure 6:**
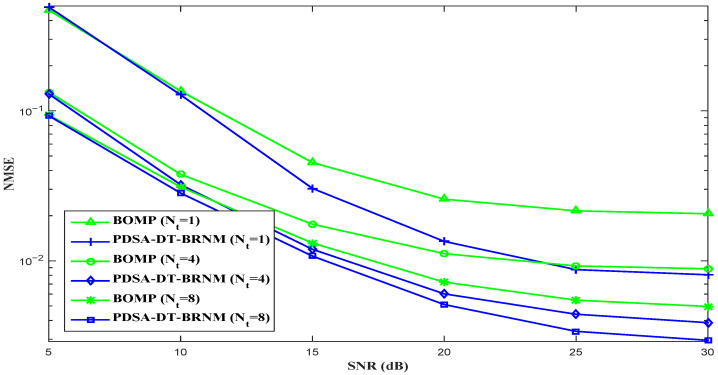
NMSE performance comparison of different algorithms with R=8 and different $N_{t}$ ($N_{t}=1,4$ or 8).

In Figure 6, we compare the NMSE performance of the proposed PDSA-DT-BRNM (R=8,D=3) algorithm and BOMP (R=8) algorithm with different $N_{t}$ ($N_{t}=1,4$ or 8). As shown in Figure 6, the proposed PDSA-DT-BRNM method achieves better performance than the BOMP algorithm considering the same number of antennas $N_{t}=1,4$ or 8, which is similar to the NMSE performance considering different *R* in Figure 3, Figure 4 and Figure 5. Moreover, both channel estimation methods can further improve the NMSE performance by employing more transmit antennas (increasing $N_{t}$); however, the proposed PDSA-DT-BRNM method can obtain more benefits from this.

## 6. Conclusions

In this paper, a novel PDSA-DT-BRNM algorithm is proposed for an MIMO-OFDM system based on the channel temporal correlation. Firstly, owing to the channel temporal correlation, whereby the previously estimated delay support and the corresponding block sparsity can be used as prior information, an efficient PDSA-DT-BRNM algorithm is proposed to iteratively choose common delay grids and estimate block gains. Then, by exploiting the intermediate channel estimation results of the first stage and the prior delay support, a new optimized channel estimation strategy is proposed. Additionally, thanks to the usage of the proposed mixed pilot arrangement strategy, the number of pilots required by the proposed method is quite limited, which is helpful in promoting the spectral efficiency. Simulation results and theoretical analysis have verified that the proposed method can achieve superior channel estimation performance to the classical BOMP method with high spectral efficiency and low computational complexity.

## Figures and Tables

**Table 1:** Parameters in different wireless communication systems.

System	Bandwidth (*B*)	Oversampled Resolvable Distance $\frac{c}{\mathrm{RB}}$
IS-95 [3]	1.25 MHz	$\frac{240}{R}$ m
3GPP LTE [3]	1.4–20 MHz	$\frac{15}{R}$–$\frac{215}{R}$ m

**Table 2:** Computational complexity comparison.

Algorithm	Computational Complexity
PDSA-DT-BRNM	$O(3MN_{t}\left(L_{cp}+R\right)K)$
BOMP	$O(MN_{t}\left(R\left(L_{cp}-1\right)+1\right)K$

**Table 3:** COST 207 TU6 channel model.

Tap Number	1	2	3	4	5	6
Delay (μs)	0.0	0.2	0.5	1.6	2.3	5.0
Power (dB)	−3	0	−2	−6	−8	−10

## Data Availability

Data is contained within the article.

## Appendix A. The Properties of ${∥\Phi_{\tau }^{H}z∥}_{2}(\tau \in T_{1})$

Based on the assumption that the *M* pilots allocated to the $i^{th}$ antenna are equispaced, $p_{m}^{\left(i\right)}=p_{0}^{\left(i\right)}+mD_{F}$, where $i=0,1,\ldots ,N_{t}-1,m=0,1,\ldots ,M-1,D_{F}=N/M,M\geq L_{cp}$, we have the expression ${∥\Phi_{\tau }^{H}z∥}_{2}$ given by [15]

(A1) $$\begin{array}{cc} {∥\Phi_{\tau }^{H}z∥}_{2}= & \sqrt{\sum_{i=0}^{N_{t}-1}{\left|h_{0}^{\left(i\right)}\sum_{m=0}^{M-1}e^{-j\frac{2\pi }{N}p_{m}^{\left(i\right)}\Delta \tau }\right|}^{2}}\left(p_{m}^{\left(i\right)}=p_{0}^{\left(i\right)}+mD_{F}\right)\\ = & \sqrt{{\left|h_{0}^{\left(0\right)}\right|}^{2}+{\left|h_{0}^{\left(1\right)}\right|}^{2}+\ldots +{\left|h_{0}^{\left(N_{t}-1\right)}\right|}^{2}}\left|\sum_{m=0}^{M-1}e^{-j\frac{2\pi }{M}m\Delta \tau }\right|\\ = & \sqrt{{\left|h_{0}^{\left(0\right)}\right|}^{2}+{\left|h_{0}^{\left(1\right)}\right|}^{2}+\ldots +{\left|h_{0}^{\left(N_{t}-1\right)}\right|}^{2}}\frac{\left|sin(\pi \Delta \tau )\right|}{\left|sin(\pi \Delta \tau /M)\right|}\\ = & \sqrt{\sum_{i=0}^{N_{t}-1}{|h_{0}^{\left(i\right)}|}^{2}}f\left(\Delta \tau \right)\left(f\left(\Delta \tau \right)=\frac{\left|sin(\pi \Delta \tau )\right|}{\left|sin(\pi \Delta \tau /M)\right|}\right) \end{array}$$

where $\left|h_{0}^{\left(i\right)}\right|$ is the amplitude of the channel tap of the $i^{th}$ antenna without leakage. The following two properties of f(Δτ) (f(−Δτ)=f(Δτ)) can be derived [15]:

(1) Within the ranges $\Delta \tau \in \left[-\frac{1}{2},0\right)$ and $\Delta \tau \in \left(0,\frac{1}{2}\right]$, f(Δτ) monotonously increases and monotonously decreases respectively.

(2) Within the ranges $\Delta \tau \in \left[L_{cp}+1,-\frac{1}{2}\right)$ and $\Delta \tau \in \left(\frac{1}{2},L_{cp}-1\right]$, we have $f\left(\Delta \tau \right)<f\left(-\frac{1}{2}\right)$ and $f\left(\Delta \tau \right)<f\left(\frac{1}{2}\right)$ respectively.
